# The antidepressant fluoxetine (Prozac®) modulates serotonin signaling to alter maternal peripartum calcium homeostasis

**DOI:** 10.1038/s41598-023-49253-4

**Published:** 2023-12-09

**Authors:** Rafael R. Domingues, Natalia N. Teixeira, Waneska S. Frizzarini, Adam D. Beard, Meghan K. Connelly, Alysia Vang, Milo C. Wiltbank, Laura L. Hernandez

**Affiliations:** 1 Department of Animal and Dairy Sciences, University of Wisconsin-Madison, 1865 Observatory Dr, Madison, WI 53706 USA; 2 Endocrinology and Reproductive Physiology Program, University of Wisconsin-Madison, Madison, WI USA; 3 Present Address: Department of Animal Sciences, The Ohio State University, Columbus, OH USA

**Keywords:** Physiology, Reproductive biology

## Abstract

Antidepressant use is two-fold greater in women compared to men; however, most studies have been performed in male subjects. We aimed to understand the impact of selective serotonin reuptake inhibitors (SSRI, most used antidepressants) on calcium homeostasis and steroid metabolism during the peripartum period. Pregnant sheep (n = 10/group) were treated with vehicle or fluoxetine (most common SSRI) during the last month of gestation. Fluoxetine treatment decreased circulating calcium prior to parturition (8.7 ± 0.1 mg/dL vs 8.2 ± 0.1 mg/dL; P = 0.07). In the control group, total calcium decreased after parturition corresponding to the onset of lactogenesis followed by increase in calcium by day 2 postpartum. Interestingly, this normal transient decrease in circulating calcium was absent in fluoxetine-treated ewes. The steroids cortisol and progesterone were not altered by fluoxetine treatment whereas estradiol was decreased after the onset of treatment (12.4 ± 1.3 vs 9.1 ± 1.2 pg/mL, P = 0.05) and prior to parturition (38.1 ± 8.1 vs 22.3 ± 4.2 pg/mL, P = 0.03). Our hypothesis was supported that fluoxetine treatment alters circulating concentrations of calcium in the peripartum period; however, we surprisingly observed a decrease in estradiol concentrations contrary to reports in in vitro studies.

## Introduction

Antidepressants are one of the most commonly used medications worldwide and selective serotonin reuptake inhibitors (SSRI) are the main class of antidepressants prescribed to pregnant women^1^. About 8–13% of pregnant women use SSRI during pregnancy^2,3^. Despite the beneficial effects of SSRI to women under diverse psychological conditions, SSRI are associated with side effects to both the mother and fetus^4^. Several studies have explored the effects of maternal use of SSRI during gestation on pregnancy outcomes and neonatal health. The use of SSRI is associated with fetal growth restriction, preterm birth, low birthweight/small for gestational age neonates, neonatal morbidity, and developmental delays among others^4–7^. However, little is known about the impacts of SSRI on maternal calcium and endocrine homeostasis during the peripartum period.

In our laboratory, we have investigated the interactions between serotonin and calcium homeostasis^8–12^. During lactation, the mammary gland synthesizes and secretes serotonin that acts within the gland to promote: (1) transport of calcium into mammary epithelial cells for milk synthesis and (2) secretion of parathyroid related protein (PTHLH) that acts on the bone to release calcium into the circulation. Serotonin’s actions have been demonstrated to be crucial for milk synthesis and to maintain calcium homeostasis as calcium demands increase due to lactogenesis. During a nonlactating state, 95% of circulating serotonin is produced by intestinal enterochromaffin cells^13,14^; however, during lactation, circulating serotonin increases and the mammary gland is the source of about 50% of total circulating serotonin^15,16^. Since SSRI increase free circulating serotonin (platelet-poor plasma) and serotonin signaling throughout the body^17^, they have the potential to alter calcium homeostasis. In mice, treatment with fluoxetine increases serotonin content and PTHLH gene expression in the mammary gland and reduces maternal trabecular bone through PTHLH-induced bone resorption by the end of the lactation period^12^. Yet, the impact of SSRI on circulating concentrations of calcium during the peripartum period is still poorly understood.

Despite the primary functions of SSRI being inhibition of serotonin transporter (SERT) in the brain, SSRI may also display off-target effects throughout the body. For example, all SSRI have been shown to modulate steroidogenesis in vitro^18–20^. Specifically, SSRI appear to increase aromatase activity and increase estrogen synthesis. In contrast, in vivo studies have shown both increased and decreased estradiol synthesis depending on study approach, pregnancy status, SSRI dose, and duration of treatment^21–23^. Little is known about the interrelationship between serotonin and estradiol, particularly during late pregnancy. The impact of SSRI on serotonin signaling and potential modulation of steroidogenesis may affect endocrine homeostasis during the peripartum period.

Due to increased serotonin signaling elicited by SSRI and its potential impact on calcium and endocrine homeostasis, we aimed to assess the effect of fluoxetine on maternal calcium regulation and endocrine profiles during the peripartum period. We hypothesized that fluoxetine treatment alters circulating calcium concentrations and increases circulating estradiol during the peripartum period.

## Results

### Fluoxetine treatment modulates circulating serotonin

Pregnancy outcome data has been previously reported^24^. Gestation length was decreased in the fluoxetine group compared to the control (147.7 ± 07 vs 152.2 ± 0.9, respectively; P = 0.0009). Accordingly, the interval between onset of experiment (E0) and parturition was longer for the control group (31.8 ± 1.0 vs 27.5 ± 0.7 days; P = 0.002).

**Figure 1:**
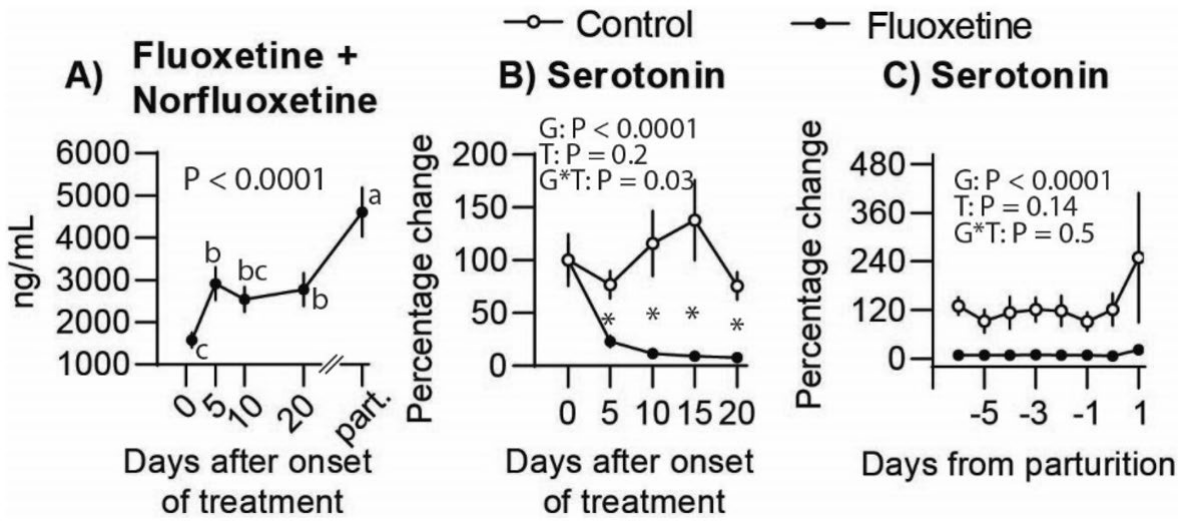
Circulating concentrations of (**A**) fluoxetine and norfluoxetine (active metabolite) and (**B**, **C**) serotonin in pregnant ewes treated with fluoxetine during the last month of gestation. In Figure (**A**), the different superscript letters indicate significant difference over time, that is, the increase in concentrations of fluoxetine + norfluoxetine in the maternal circulation. *P ≤ 0.05 between groups on each day.

Circulating fluoxetine concentrations were undetectable in the control group. In the fluoxetine group, circulating fluoxetine 24 h after the first treatment ranged from 389 to 2700 ng/mL (Fig. 1A). At parturition (P0), and cessation of treatment, circulating fluoxetine concentrations ranged from 1262.3 to 6015.3 ng/mL. Overall, fluoxetine concentrations were increased at parturition compared to previous days. There were no differences in serum serotonin concentrations between groups prior to the onset of treatment (1690.3 ± 420.4 ng/mL control and 2312.2 ± 377.3 ng/mL fluoxetine; P = 0.29). Overall, from E5 to 20 there were significant treatment effect and treatment by time interaction, while from P–6 to P0 there was a treatment effect (Fig. 1B,C). Serum serotonin was increased in the control group compared to fluoxetine. In the fluoxetine-treated ewes, serotonin concentrations were decreased by E5 (P = 0.0002) with a further decrease to E10 (P = 0.057) followed by a plateau until parturition. Between the day of parturition (P0) and 6 days postpartum (P6), the percentage increase in serum serotonin was similar (P = 0.9) between the control group (82%; 1212.3 ± 300.3 vs 2586.6 ± 835.7 ng/mL) and fluoxetine (95%; 181.0 ± 77.8 vs 483.7 ± 153.5 ng/mL) groups.

### Fluoxetine treatment alters peripartum calcium homeostasis

**Figure 2:**
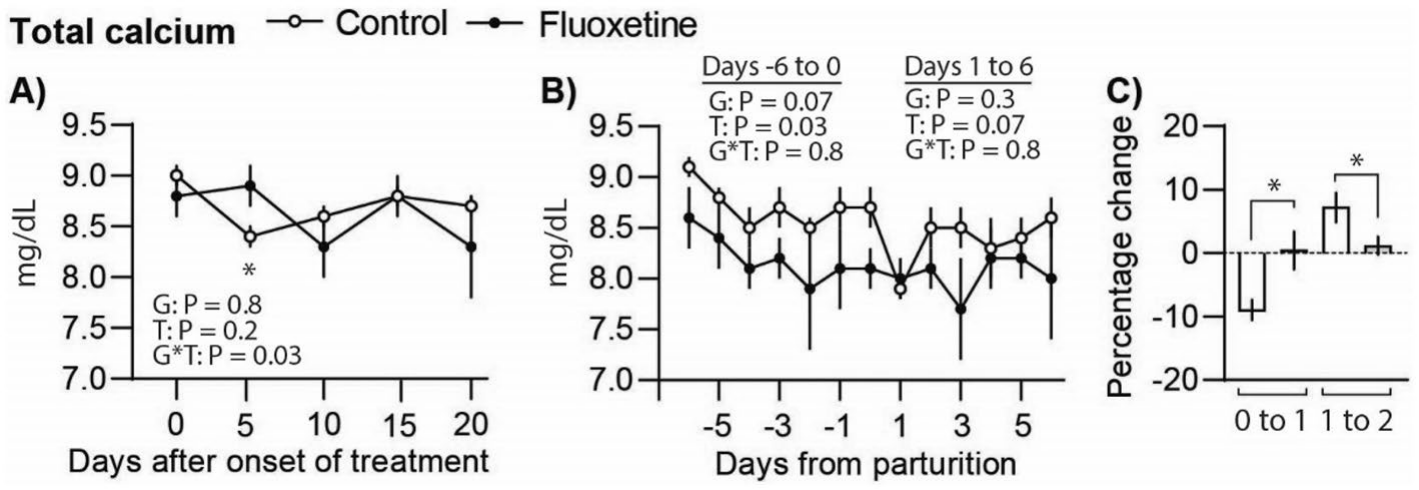
Total concentrations of calcium in pregnant ewes treated with fluoxetine during the last month of gestation (**A**, **B**). (**C**) Change in concentrations of total calcium between postpartum days 0–1 and 1–2. *P ≤ 0.05 between groups.

Circulating calcium concentrations were similar between groups before onset of treatment (9.0 ± 0.1 mg/dL control and 8.8 ± 0.2 mg/dL fluoxetine; P = 0.4) and overall from E5 to E20 (Fig. 2A). Calcium concentrations decreased in the control group only on E5. From P–6 to P0, total calcium tended to be decreased in the fluoxetine group compared to the control (Fig. 2B). From P1 to P6, total calcium concentrations were not different between groups. To further explore the impact of fluoxetine treatment on calcium homeostasis, we explored the transient decrease in calcium associated with onset of milk production around parturition. Total calcium concentrations decreased in the control group from P0 to P1 (8.7 ± 0.2 vs 7.9 ± 0.1 mg/dL, respectively; P = 0.006) followed by increased calcium concentrations by P2 (8.5 0.2 mg/dL; P = 0.01). No significant changes in calcium concentrations in fluoxetine-treated ewes was observed surrounding parturition. This is further supported by the percentage change in circulating calcium between P0–P1 and P1–P2 (Fig. 2C). Calcium concentrations in the colostrum (P0) and milk (P6) were not different between groups^24^.

### Fluoxetine treatment modulates mammary gland

**Figure 3:**
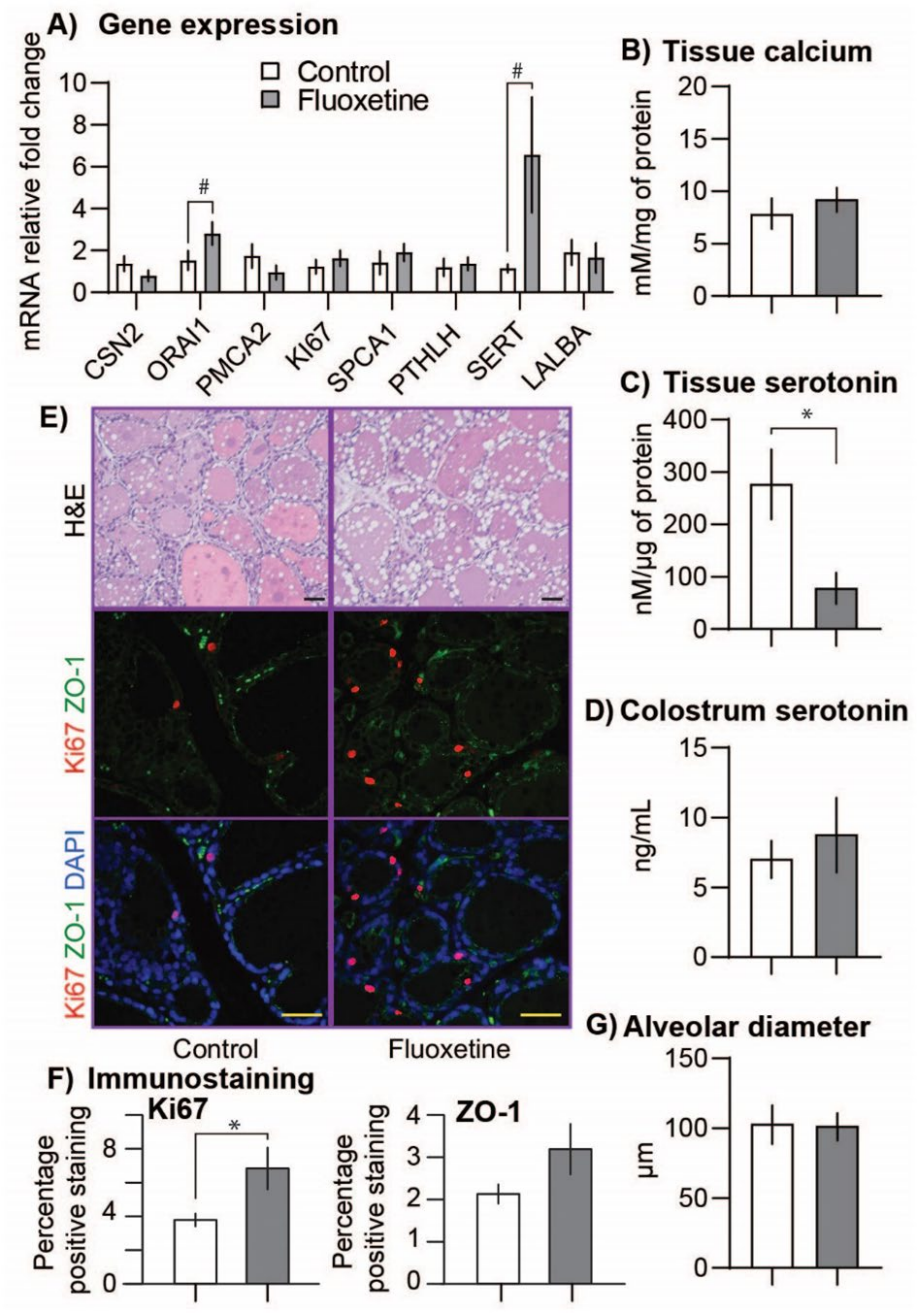
Effect of fluoxetine treatment during the last month of gestation on the ovine mammary gland. (**A**) Expression of mRNA for gene related to serotonin metabolism and calcium transport. (**B**) Mammary gland content of total calcium. (**C**) Mammary gland content of serotonin. (**D**) Colostrum serotonin concentrations. (**E**) Mammary gland histology. H&E, Ki67, and zonula occludens-1 (ZO-1) staining, DAPI. Bar = 50 µm. (**F**) Relative percentage area of positive staining for Ki67 and ZO-1 by the area of DAPI. (**G**) Mammary gland alveolar diameter. *P ≤ 0.05 and ^#^0.05 < P ≤ 0.1 between groups. Mammary gland biopsy was collected on experimental day 20.

Mammary gland gene expression is shown in Fig. 3A. Only calcium release-activated channel protein 1 (ORAI1) and serotonin transporter (SERT) tended to be increased in the fluoxetine-treated ewes. Mammary gland calcium content was not different among groups (Fig. 3B). Total concentrations of serotonin in the mammary gland tissue were decreased in the fluoxetine group (Fig. 3C) but not altered in the colostrum (Fig. 3D). Immunostaining for Ki67, a proliferation marker, was greater in the fluoxetine group. Immunostaing for ZO-1, a tight junction protein, was not different between groups (Fig. 3E,F). Mammary alveolar diameter was not different among groups (Fig. 3E–G).

### Fluoxetine treatment modulates estradiol concentrations but not other steroids

**Figure 4:**
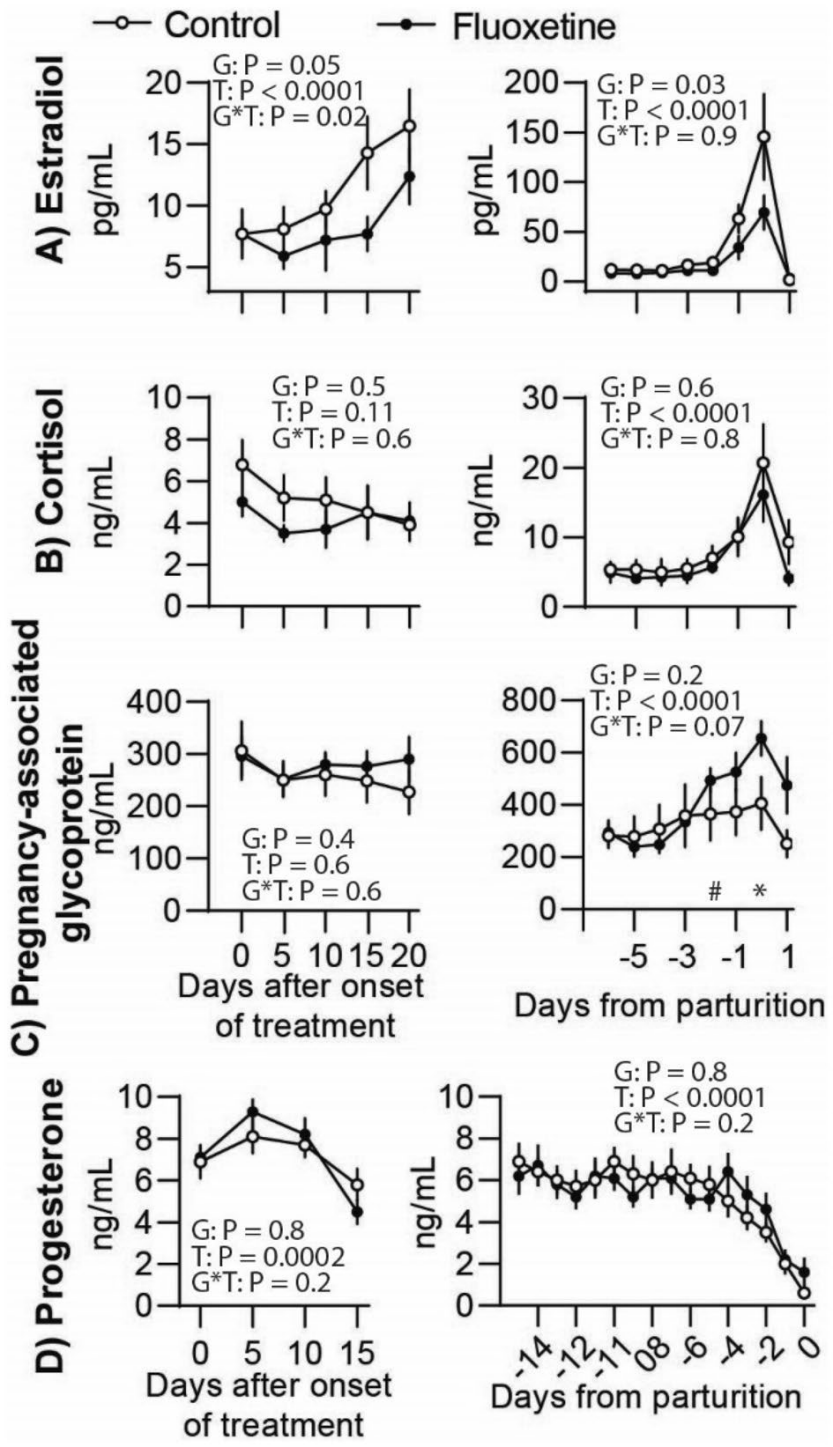
Effect of fluoxetine treatment during the last month of gestation on (**A**) estradiol, (**B**) cortisol, (**C**) pregnancy-associated glycoprotein, and (**D**) progesterone. *P ≤ 0.05 and ^#^0.05 < P ≤ 0.1 between groups on each day.

There were no differences in estradiol concentrations between groups before onset of treatment (7.7 ± 1.0 pg/mL control and 7.7 ± 2.0 pg/mL fluoxetine; P = 0.29) (Fig. 4A). From E5 to E20, estradiol was decreased in the fluoxetine group compared to the control. From days P–6 to P0, decreased estradiol concentrations were observed in the fluoxetine group. Overall, in both the control and fluoxetine groups, estradiol increased on P–1 and P0 followed by a decrease on P1. Cortisol concentrations were not different between groups from E5 to E20 and P–6 to P0 (Fig. 4B). A significant time effect from P–6 to P0 indicates cortisol increased on P–1 with a peak on P0 followed by a decrease on P1. Concentrations of pregnancy-associated glycoproteins (PAG) were similar between groups from E0 to E20 (Fig. 4C). From P-6 to P0 there was a significant time effect with maximal PAG observed on P0 followed by a decrease on P1. Concentrations of PAG tended to be increased on days P-2 and P0 in the fluoxetine group. Progesterone concentrations were not different between groups (Fig. 4D). Overall, progesterone began to decline between P–4 and P–2 followed by continuous decrease to P–1 and P0, the day with lowest circulating progesterone.

## Discussion

As the use of antidepressants has increased over the last several decades, comprehensive understanding of their off-target, nonneuronal effects is critical, particularly in pregnant and lactating women. Our study was focused on the use of SSRI during the peripartum period to determine the effects of fluoxetine on calcium and endocrine homeostasis. Additionally, from a physiological perspective, this study aids to underpin the endocrine interactions between serotonin, calcium, and estradiol. Taken together, we demonstrated that fluoxetine treatment alters calcium homeostasis and decreases circulating estradiol during the peripartum period.

In the bloodstream, serotonin is primarily transported inside platelets after uptake by the SERT located on the platelet plasma membrane^13,14,25^. Thus, platelet SERT is a key regulator of free circulating concentrations of serotonin^14,17^. Inhibition of platelet SERT by SSRI prevents platelet uptake of serotonin resulting in decreased serum concentrations of serotonin as observed in the present study. However, the SSRI inhibition of platelet SERT results in increased free circulating serotonin due to platelet inhibition of regulating free amounts of the hormone^4,17^. Interestingly, serum serotonin increased in both control and fluoxetine groups after parturition. The increase in serotonin is likely due to mammary gland secretion of serotonin in the control ewes^12,16^ while in the fluoxetine group it is likely due to upregulation of mammary gland synthesis of serotonin in combination with cessation of fluoxetine treatment and initial reestablishment of total (intraplatelet + free) serotonin content in the circulation.

Treatment with 5HTP (serotonin precursor) promotes transient reduction in circulating calcium concentrations in lactating dairy cows resulting in a hypercalcemic compensation post-treatment^8^. It is likely that the decreased calcium concentrations are due to increased calcium transport into the mammary gland. Previous work in our laboratory has demonstrated that serotonin upregulates calcium transporters (i.e. ORAI1) in the mammary epithelium, resulting in greater calcium content in the mammary gland tissue and in the milk in cattle and rodents^8,9,26,27^. In our ovine model of fluoxetine treatment, increased serotonin due to fluoxetine treatment was associated with decreased maternal calcium for the week prior to parturition (present study) and decreased neonatal calcium during the week postpartum^24^. However, in contrast to the increased transport of calcium into the mammary gland due to increased serotonin in lactating dairy cows^8,27^, in the pregnant nonlactating sheep in the present study, increased serotonin signaling was not associated with increased mammary gland calcium content. Given that serotonin promotes upregulation of calcium transporter (including ORAI1) in other tissues^28–30^ thereby regulating calcium transport into cells, the decrease in circulating calcium in ewes and lambs may be associated with increased calcium uptake in multiple tissues. In mice, fluoxetine increased calcium at the end of lactation^12^ whereas sertraline (currently the most used SSRI) decreased circulating calcium during the peripartum period^31^ similarly to the effect of fluoxetine in sheep. Additionally, sertraline-induced increase in serotonin was associated with increased expression of calcium transporters in the intestines and greater kidney calcium content (likely increasing intestinal absorption and decreasing kidney excretion of calcium) as a homeostatic mechanism to counterbalance the serotonin-induced decrease in circulating calcium.

An interesting finding in the present study was the effect of fluoxetine on the dynamics of circulating calcium in the first days postpartum. With the onset of lactogenesis around the time of parturition, mammary gland demands for calcium rapidly rise resulting in a transient decrease in circulating calcium on the day after parturition^15,32,33^. The decrease in circulating calcium activates endogenous mechanisms for restoration of circulating concentrations of calcium typically by day 2 postpartum. Mechanisms of calcemic adaptations to lactation include bone resorption, increased gastrointestinal absorption, and renal reabsorption mediated by classic endocrine signals (parathyroid hormone, calcitonin, and 1,25-dihydroxyvitamin D) and mammary gland-derived endocrine signals (serotonin and PTHLH)^32,34,35^. The normal dynamics of circulating calcium homeostasis during the peripartum period is observed in the control group whereas the transient decrease in calcium did not occur in the fluoxetine group. We believe that the serotonin-mediated fluoxetine-induced decrease in circulating calcium prior to parturition promoted activation/upregulation of the endogenous mechanisms to increase circulating calcium allowing for enhanced adaptation to lactation. On a similar note, lactating cows have more robust response to calcium perturbations than nonlactating cows given that multiple mechanisms of calcemic adaptations are more readily activated during lactation^15^.

The impact of SSRI treatment on estradiol is complex. Although several in vitro studies have demonstrated that SSRI increase aromatase activity and estradiol synthesis^18–20,36,37^, in vivo studies have conflicting results with increased or decreased estrogen synthesis and signaling^23^. However, most studies have been conducted in male subjects disregarding the effect of sex. In females, it appears that in vivo short-term fluoxetine treatment increases systemic concentrations of estrogen^22^ whereas long-term treatment appears to reduce it^21,38,39^. In the present study, fluoxetine treatment decreased estrogen concentrations shortly after onset of treatment until the day of parturition. The impacts of increased serotonin signaling on estrogen metabolism is poorly understood; however, some pregnancy pathologies (gestational diabetes mellitus, preeclampsia, and hyperemesis gravidarum) that are associated with increased serotonin display decreased circulating estrogens supporting a possible role for serotonin on estrogen metabolism^37^. As the placenta is the main source of estrogen during late pregnancy in sheep, the decrease in estrogen in the fluoxetine group in the present study could be related to decreased placentome growth and vascular perfusion^24^ limiting placental capacity to synthesize estrogens. Accordingly, the present finding may be relevant only during gestation and not represent the impact of SSRI exposure in nonpregnant individuals. An important aspect that remains to be elucidated, particularly in females, is whether the short- and long-term in vivo interrelationship among SSRI, serotonin, and steroidogenesis occur at the neuronal (central) or peripheral levels. Another important aspect related to SSRI use, circulating estrogen, and calcium metabolism is bone metabolism. Fluoxetine treatment has been associated with decreased bone mineral density and increased risk for bone fracture in humans and rodents^12,40,41^. Although a direct serotonin-induced mechanism decreasing bone mineral density has been demonstrated^42^, estrogen also plays an important role on bone metabolism^43^. For example, decreased estrogen after menopause is associated with decreased bone mineral density and increased risk for bone fractures. Decreased estrogen due to SSRI treatment may enhance the effects of SSRI-induced increased serotonin leading to decreased bone mineral density and greater risk for bone fractures as observed in humans taking SSRI. Further research in female subjects is warranted as the use of SSRI is two-fold in women compared to men and bone loss is greater in postmenopausal women.

In our previous report demonstrating the effects of SSRI on pregnancy outcomes in sheep, we demonstrated decreased placentome (function unit of placenta in sheep) growth during the last month of gestation associated with fluoxetine treatment^24^. As placental growth is associated with its blood perfusion^44^, we speculate whether decreased estradiol observed in the present study is associated with decreased placental perfusion/growth and fetal growth. Despite a clear role of serotonin decreasing uterine and placental vascular perfusion by promoting vasoconstriction of uterine/placental vascular beds^4,45–47^, decreased estrogen may also be involved in the decreased perfusion and growth of the placenta as estradiol regulates uteroplacental angiogenesis, endothelial cell proliferation, and vasodilation through genomic and nongenomic pathways^48–50^. Further studies are needed to unravel the role of serotonin and estradiol on placental blood perfusion and development in human and animal models treated with SSRI.

A weakness of our study is that our model did not include a group with depression or other psychological conditions. The underlying psychological condition itself affect maternal endocrine homeostasis as maternal depression is associated with altered hypothalamus–pituitary–adrenal regulation^51,52^. Increased maternal stress and circulating cortisol due to depression during pregnancy can affect fetal response to stress which is critical for initiation of labor^53^. In the present study, fluoxetine treatment was initiated in the last month of gestation which is not common in women as most women taking untidepressants during gestation initiate treatment prior to pregnancy establishment^7^. Nevetheless, SSRI exposure during the third trimester of gestation is associated with more adverse effects on neonatal health^2,3,24,54^. We did not assess whether the gestational period of onset of antidepressant treatment affects endocrine and calcium hosmeostasis regulation.

In conclusion, fluoxetine treatment in pregnant sheep increases serotonin signaling with modulation of calcium homeostasis and estrogen metabolism. Interestingly, hypocalcemia prior to parturition in fluoxetine-treated ewes appears to enhance lactogenic responses to calcium perturbations preventing the transient decrease in calcium associated with the onset of lactogenesis. The endocrine mechanisms for SSRI-induced decreased circulating estrogens remains to be elucidated.

## Materials and methods

### Animal management

All methods were carried out in accordance with the University of Wisconsin-Madison IACUC guidelines under the approved protocol #A006302-A02. Further, appropriate ARRIVE 2.0 guidelines from the National Centre for the Replacement, Refinement, and Reduction of Animals in research published in PLOS Biology were strictly followed.

Timed-bred multiparous Hampshire ewes (4.4 ± 0.4 years old) were obtained from the Arlington Sheep Research Unit from the University of Wisconsin-Madison. Beginning on day 112 ± 1 postbreeding, 20 pregnant ewes were housed in individual pens at the Livestock Laboratory at the University of Wisconsin-Madison and maintained at a constant temperature at 18 °C and a 14/10 h light/dark cycle. All ewes received ad libitum access to water and were individually fed haylage, whole-shell corn, and mineral supplement based on live weight according to National Research Council Nutrient Requirements for pregnant ewes^55^.

On day 117 ± 1 postbreeding, jugular catheters were placed in all ewes for intravenous treatment and blood collection. The catheter was aseptically placed, fixed to the neck and protected by a bandage. Catheter patency was maintained during the entire period of treatment/ blood collection.

Ewes were pregnant with a range of one to three fetuses; the average number of fetuses was not different between groups (P = 0.5; 1.6 ± 0.2 vs 1.8 ± 0.2). Delivery was assisted as needed and lambs were fed colostrum by assisted nursing or bottle-fed fresh colostrum from their dam.

### Experimental design

**Figure 5:**
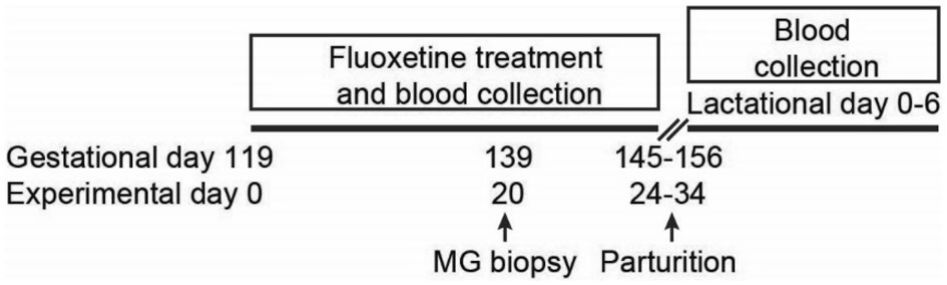
Experimental design indicating key period for fluoxetine treatment and sample collection. Treatment was initiated on experimental day 0 (gestational day 119 ± 1). Mammary gland (MG) biopsy was collected on experimental day 20. Blood sample from ewes were collected daily throughout the experimental period (gestation and lactation).

On day 119 ± 1 postbreeding (experimental day 0; E0) of a 151 day expected gestation, ewes were randomly assigned to control or fluoxetine groups. Fluoxetine-treated ewes received fluoxetine hydrochloride (C845, AK Scientific, Union City, California, USA) at 10 mg/kg on experimental day (E) E0 and E1 and at 5 mg/kg daily thereafter until parturition. Fluoxetine dosage was based on a previous experiment from our laboratory aiming to be representative of systemic fluoxetine concentrations in humans (Fig. 5A). Lyophilized fluoxetine was reconstituted daily in ethanol and diluted with 0.9% NaCl saline (07983-02, Hospira, Lake Forest, Illinois, USA) to the appropriate concentration for each ewe based on body weight. Final ethanol concentration was < 3.5%. Body weight was assessed weekly and fluoxetine dose was adjusted accordingly. Ewes in the control group received saline + ethanol at similar ethanol concentration to fluoxetine-treated ewes. All ewes were treated at a continuous infusion rate of 200 mL for 15 min using an automated mini pump (Heska Vet/IV 2.2, Heska, Loveland, Colorado, USA).

### Blood and colostrum collections and hormone analysis

Blood samples were collected from jugular catheters immediately before each treatment during the prepartum period and for 6 days postpartum. For serum samples, blood was collected into evacuated tubes. Tubes containing EDTA were used for obtaining plasma. Blood samples were centrifuged at 2000 × *g* for 15 min and serum/plasma were stored at  − 20 °C until assayed. Colostrum was collected within 30 min of partur ition and before lamb intake.

Serum concentrations of serotonin were determined by EIA (IM1749, Beckman Coulter, Czech Republic) according to manufacturer instructions; serum was diluted 1:100 and colostrum was diluted 1:5. The intra- and inter-assay CV were 4.2% and 7.4%, respectively. Plasma estradiol was determined by RIA (Ultra Sensitive Estradiol DSL-4800 Beckman Coulter, Prague, Czech Republic) per manufacturer instructions; the intra-assay CV was 7.4%. Plasma progesterone was determined by RIA (ImmuChem Coated Tube Progesterone 125I RIA Kit, MP Biomedicals, CA, USA) per manufacturer instructions; the intra-assay CV was 7.9%. Plasma cortisol was determined by EIA as validated in our laboratory^56^. The intra- and inter-assay CV were 3.5% and 4.5%, respectively. Plasma pregnancy-associated glycoprotein was determined by EIA (BioPRYN Flex; BioTracking LLC, Moscow, ID). The intra- and inter-assay CV were 6.7% and 11.0%, respectively. Total serum calcium concentrations were determined using Catachem reagents (C294-50, Oxford, Connecticut, USA) on a ChemWell-T analyzer.

### Mammary gland biopsy and analysis

On experimental day 20 (day 139 ± 1 postbreeding) a mammary gland biopsy was collected as described^27^. A fragment of tissue was immediately frozen in liquid nitrogen for later RNA extraction and measurement of total calcium while another fragment was placed in 4% paraformaldehyde for 24 h followed by storage in 70% ethanol until histological processing.

To obtain RNA from mammary gland, tissue was homogenized using Bullet Bender Bead lysis kit (GREENE1, Next Advance, Troy, NY, USA) on Bullet Bender Tissue 24 (Next Advance, Troy, NY, USA) followed by RNA extraction with Quick-RNA MiniPrep Plus kit (Zymo Research, Irvine, CA, USA) per manufacturer instruction. Concentrations of RNA were determined by spectrometry with a NanoDrop 2000 spectrophotometer (Thermo Scientific, Waltham, MA, USA). Complementary DNA (cDNA) was synthesized using the High-Capacity cDNA Reverse Transcription Kit (Applied Biosystems, Foster City, CA, USA) as described by the manufacturer using 1 μg of total RNA. The cDNA was used directly for quantitative real-time PCR (qRT-PCR). The qRT-PCR reactions were carried out on an CFX Connect Real-Time PCR system (Bio-Rad Life Science, Hercules, CA, USA) using a master mix containing a total volume of 10.5 μL per tube consisting of 6.25 μL of SsoFast EvaGreen Supermix (Bio-Rad Life Science, Hercules, CA, USA), 3.25 μL of nuclease-free water, and 0.5 μL of forward and reverse primers (10 μM), and 2 μL of cDNA at a 1:4 dilution were added to the master mix for a total reaction volume of 12.5 μL. All samples were analyzed in duplicate. The reactions were initiated with preincubation at 95 °C for 3 min followed by 42 cycles of denaturation (95 °C for 10 s) and annealing and extension (60 °C for 30 s). The primer sequences for targeted genes (Table 1) were synthesized by Integrated DNA Technologies Inc. (San Diego, CA, USA). Efficiencies of qRT-PCR for amplification of targeted genes were determined in our laboratory and ranged from 93 to 108%. The amplification data obtained from the qRT-PCR as the cycle threshold (Ct) were used to calculate the mRNA relative abundance of each sample by the 2^−ΔΔCt^ method^57^ using the control group as baseline; therefore, samples from the fluoxetine group are represented by their relative fold-change from the control group. The geometric means of UCB and GAPDH genes were used as housekeeping genes.

**Table 1:** Primer sequences for quantitative real-time PCR.

Gene	Forward	Reverse
ORAI1	GATGAGCCTCAACGAGCACT	TGTACCTCCACCATTGCCAC
PTHRP	AGGGTGTGTGGAGTCAACTTT	CTGGGGACCTAGCTGTCTGTC
SERT	CGAAGTGGCCAAAGATGCAG	GGCAAAGAATGTGGATGCCG
CSN2	ATCGAGAGCCATGAAGGTCC	GCTTTCCACAGTCTCACCGA
MKI67	TGAAAGTTAGGGTGTCCGCC	GAGACTCTCCACGTGCAACA
LALBA	CCCCTTGGCTACCTCGTTTT	ACCGAGCAAGGGTCAAATGT
PMCA2	GGATGCCTTCAGCTACCAG	GCGTCTTGCTGTTTGGCTTT
GAPDH	GGCGTGAACCACGAGAAGTA	GGCGTGGACAGTGGTCATAA
SPCA1	TTCATGTGGTTGCTGACAGG	GTGCAACCTGTTCTTCCTCTCT
UCB	CGTCTTAGGGGTGGCTGTTA	AAATTGGGGTAAATGGCTAGA

Histology samples were embedded in paraffin, sectioned into 8 μm sections, stained with conventional hematoxylin–eosin and observed by light microscopy for image collection and analyzed qualitatively by a single technician unaware of treatment group. For immunofluorescence, slides were processed as described^31^. Ki67 (1:200; ab15580, ABCAM, Boston, MA, USA) and zonula occludens-1 (ZO-1; 1:100; 33-9100, Invitrogen, Eugene, OR, USA) were used as primary antibodies. The following secondary antibodies were used: AlexaFluor 594 (A11012, goat anti-rabbit) and 488 (A11001, goat-anti-mouse) from Life Technology at a 1:250 dilution. Nuclei were counterstained with DAPI (1:1500; D3571, Invitrogen, Eugene, OR, USA). Images were obtained with a Zeiss AX10 microscope and images captured with a Basler acA2440-35ucMED camera. Manual WSI software (Microvisioneer, Germany) was used for image acquisition. Images were analyzed on ImageJ software. The relative percentage area of positive staing for Ki67 and ZO-1 were determined based on the total area of DAPI staining.

Total protein in the mammary gland tissue was immunoprecipitated with Triton buffer and Halt protease and phosphatase inhibitor cocktail (1861284, Thermo Scientific, Rockford, IL, USA). Total protein concentrations were determined by BCA assay (K813-5000, BioVision, Milpitas, CA, USA). Total calcium content in the mammary gland was determined as described^15^ using 75 µg of mammary gland protein for the assay (701220, Cayman Chemical Company, Ann Arbor, MI, USA). Total serotonin content in the mammary gland was determined as described^15^ with 50 µg of mammary gland protein used in the assay (IM1749, Beckman Coulter, Czech Republic).

### Statistical analysis

All statistical analysis was performed using SAS (version 9.4; SAS Institute Inc., Cary, North Carolina, USA). Data were analyzed with PROC MIXED procedure using one-way ANOVA and two-way ANOVA for repeated measures. Tukey HSH was used for post hoc comparisons. Studentized residuals with deviations from assumptions of normality and/or homogeneity of variance were transformed into square root, logarithms, or ranks. A probability of ≤ 0.05 indicated a difference was significant and a probability between > 0.05 and ≤ 0.1 indicated significance was approached. Data are presented as the mean ± standard error of mean (SEM).

## Data Availability

The data underlying this article are available in the article.
